# Inhibition of Atrogin-1/MAFbx Mediated MyoD Proteolysis Prevents Skeletal Muscle Atrophy *In Vivo*

**DOI:** 10.1371/journal.pone.0004973

**Published:** 2009-03-25

**Authors:** Julie Lagirand-Cantaloube, Karen Cornille, Alfredo Csibi, Sabrina Batonnet-Pichon, Marie Pierre Leibovitch, Serge A. Leibovitch

**Affiliations:** 1 Laboratoire de Génomique Fonctionnelle et Myogenèse, UMR866 Différenciation Cellulaire et Croissance, INRA UM II, Campus INRA/SupAgro, Montpellier, France; 2 EA 300, Stress et Pathologies du Cytosquelette, Paris, France; McMaster University, Canada

## Abstract

Ubiquitin ligase Atrogin1/Muscle Atrophy F-box (MAFbx) up-regulation is required for skeletal muscle atrophy but substrates and function during the atrophic process are poorly known. The transcription factor MyoD controls myogenic stem cell function and differentiation, and seems necessary to maintain the differentiated phenotype of adult fast skeletal muscle fibres. We previously showed that MAFbx mediates MyoD proteolysis *in vitro*. Here we present evidence that MAFbx targets MyoD for degradation in several models of skeletal muscle atrophy. In cultured myotubes undergoing atrophy, MAFbx expression increases, leading to a cytoplasmic-nuclear shuttling of MAFbx and a selective suppression of MyoD. Conversely, transfection of myotubes with sh-RNA-mediated MAFbx gene silencing (shRNAi) inhibited MyoD proteolysis linked to atrophy. Furthermore, overexpression of a mutant MyoDK133R lacking MAFbx-mediated ubiquitination prevents atrophy of mouse primary myotubes and skeletal muscle fibres *in vivo*. Regarding the complex role of MyoD in adult skeletal muscle plasticity and homeostasis, its rapid suppression by MAFbx seems to be a major event leading to skeletal muscle wasting. Our results point out MyoD as the second MAFbx skeletal muscle target by which powerful therapies could be developed.

## Introduction

Skeletal muscle atrophy is characterized by an increase in proteolysis, particularly via the ATP-dependent ubiquitin-proteasome pathway [1]. One of the proteins induced most dramatically in numerous models of atrophy is the muscle-specific F-box MAFbx [2], [3] that associates with Skp1, Cul1 and Roc1 to form an SCF-type ubiquitin ligase [4], [5]. Overexpression of MAFbx in skeletal myotubes leads to atrophy, whereas knockdown of *MAFbx* in mice leads to significant resistance to skeletal muscle denervation atrophy [2]. Furthermore, F-box proteins confer SCF specificity by directly interacting with the substrate and there are increasing evidence about MAFbx participation in a muscle-specific ubiquitin ligase complex leading to negative regulation of muscle cell size [6], [7]. However, the mechanisms by which MAFbx contributes to atrophy remain unclear. Recently, we have shown that overexpression of MAFbx in proliferating myoblasts antagonizes differentiation by inducing MyoD degradation [8]. MyoD is a muscle-specific transcription factor that induces both the withdrawal from the cell cycle and the activation of muscle specific genes expression crucial for skeletal muscle differentiation process engagement [9]–[11]. MyoD is also essential for myogenic stem cell function in adult skeletal muscle [12]. Moreover, MyoD is normally expressed in adult fibres where its protein levels tend to increase with development and remain relatively constant during aging *in vivo* [13], [14]. In muscle fibrenuclei, MyoD seems also required to maintain aging muscle homeostasis and plays a role in skeletal muscle plasticity in response to hypertrophic or denervation stimuli [13], [15], [16]. Therefore, in atrophic conditions, MAFbx-dependent proteolysis of MyoD could constitute a major event to suppress muscle homeostasis. To address this problem, in the present work we have used various *in cellulo* and *in vivo* muscle atrophy models to examine the effects on MyoD degradation during the atrophic process. we present evidence that MyoD is targeted by Atrogin1/MAFbx (MAFbx) in skeletal muscle atrophy. In cultured myotubes undergoing atrophy, the expression of MAFbx increases, leading to a cytoplasmic-nuclear shuttling of MAFbx and degradation of MyoD. Among the four MRFs, MyoD was selectively affected as confirmed by MyoD over-ubiquitination. Conversely, transfection of myotubes undergoing atrophy with shRNA-mediated MAFbx gene silencing (shRNAi) prevented MyoD degradation. Finally, overexpression of a MyoD mutant (K133R) lacking MAFbx-mediated ubiquitination, not only reduced starvation-induced muscle atrophy in mouse primary cultures of myotubes and in mice but lead to a hypertrophy in control muscle. These results suggest that the targeting of MyoD by MAFbx may be a major event to suppress the complex role of MyoD in plasticity and homeostasis in skeletal muscle. Moreover, the maintain of MyoDK133R in muscle undergoing atrophy has a protective effect against further wasting. MyoD K133R represents a new pharmacological target to limit muscle atrophy, in a profilatic or curative perspective.

## Results

### MyoD but not the others MRFs interacts with MAFbx

MAFbx contains two potential nuclear localization signals which both are conserved between human, rat and mouse species [3], [8] suggesting that during muscle atrophy MAFbx might ubiquitinate muscle-specific transcription factors or nuclear proteins involved in muscle growth. Indeed, we provided evidence that ectopically expressed MAFbx interacts with MyoD but not Myf5 in myoblasts [8]. This prompted us to test the interaction of MAFbx with the two other muscle specific transcription factors myogenin and MRF4. We performed co-immunoprecipitation experiments. 10T1/2 cells were co-transfected with HA-tagged MyoD, myogenin, MRF4 and Flag-MAFbx expression constructs. Cell extracts were subjected to immunoprecipitation with anti-Flag antibodies, followed by immunoblotting analysis with anti-HA antibodies. MyoD but neither myogenin nor MRF4 coimmunoprecipitated with MAFbx (Supplementary data Fig S1). These data show that among the four MRFs, MyoD is the only one that interacts with MAFbx.

### Increasing nuclear localization of MAFbx in C2C12 myotubes that undergo atrophy

**Figure 1:**
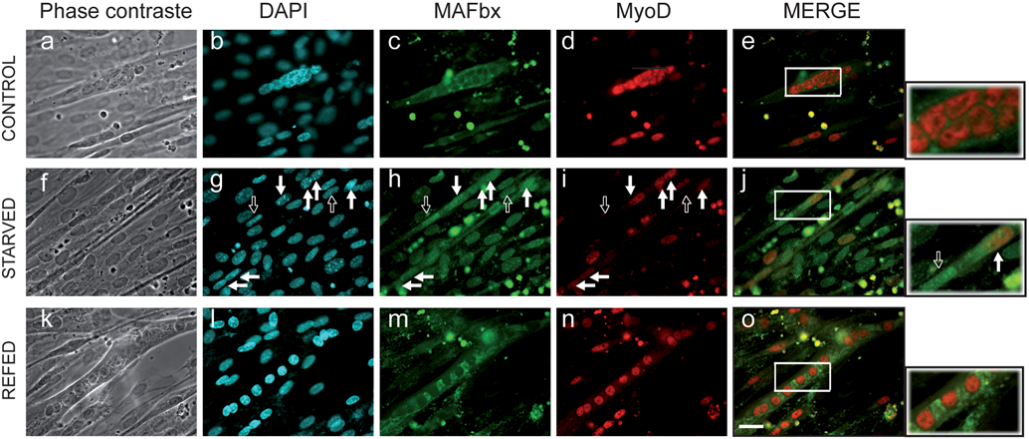
Starvation induces nuclear localization of the MAFbx protein and degradation of MyoD in C2C12 myotubes. C2C12 myotubes at day4 of differentiation were starved by removal of growth medium, amino acids and glucose and were incubated in PBS for 6 h (Starved). Fresh medium, amino acids and glucose were replaced in the refed culture for 15 h (Refed). Myotubes were fixed and immunostained with anti-MAFbx polyclonal antibodies and anti-MyoD monoclonal antibodies (5.8A). DNA was stained by DAPI. Myotubes at 4 days of differentiation were used as control. (Magnifications are ×400). Empty arrows indicate a nuclear localization of MAFbx and low levels of MyoD in atrophic myotubes. Full arrows show MyoD degradation in atrophying myotubes. The right panels show enlarged viewed of the boxed areas.

**Figure 2:**
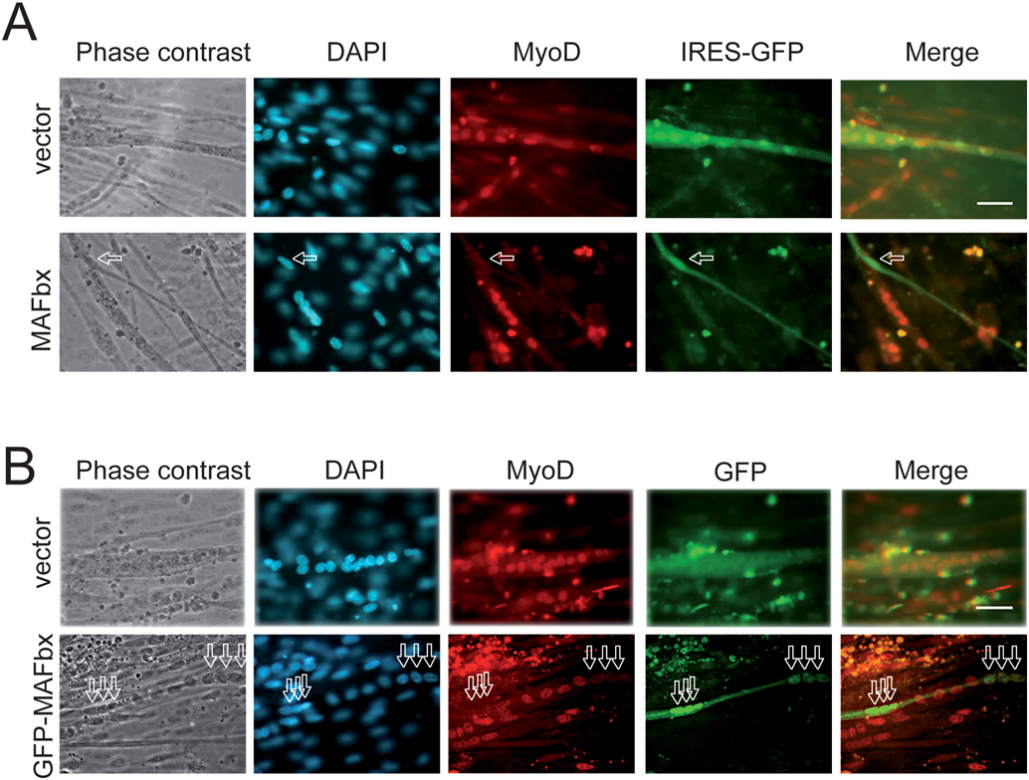
MAFbx overepression suppresses MyoD in C2C12 myotubes. A/ C2C12 myotubes were transfected with either empty pcDNA4-IRES-GFP vector or MAFbx-IRES-GFP. Twenty-four hours later, myotubes were fixed, stained with anti-MyoD (5.8A) and revealed with goat anti-mouse antibody conjugated to Texas Red. DAPI shows nuclei. Arrows indicate that overexpression of MAFbx (marked by the green GFP) in myotubes induces an atrophic phenotype and the loss of MyoD staining (absence of red myonuclei). Images of a representative field were obtained by indirect immunofluorescence microscopy. Magnifications are ×400. B/ Overexpression of the fusion GFP-MAFbx localizes in the nuclei of myotubes and suppresses MyoD expression. C2C12 myotubes were transfected either with pCMV-GFP expression plasmid (vector) or pCMV-GFP-MAFbx (MAFbx bound to GFP). Twenty- four hours later, myotubes were fixed and stained with anti-MyoD antibodies as in (A) and with DAPI. Arrows indicate that overexpression of MAFbx (marked by the green GFP) in the nuclei of myotubes. Images of a representative field were obtained by indirect immunofluorescence microscopy. Magnifications are ×400.

Overexpression of MAFbx in proliferating myoblasts antagonizes differentiation, inducing nuclear MyoD degradation and preventing muscle-specific-gene activation [8]. MAFbx has also been suggested to interact with cytoplasmic proteins such as calcineurin A and α-actinin-2 at the Z-disc in cardiomyocytes [17]. Altogether these data prompted us to investigate the cellular localization of MAFbx in skeletal muscle atrophy conditions. As food deprivation leads to rapid muscle wasting and increases MAFbx mRNA expression *in vivo* and in C2C12 cultures [6], [7] we repeated this experiment to follow MyoD immuno-staining. In control myotubes, MyoD showed a typical nuclear staining while low levels of cytoplasmic MAFbx were observed (Fig. 1, a–e). After 6 hours of starvation, myotubes showed a 50–60% decrease in diameter [7], a loss of myonuclei and a nuclear localization of MAFbx. In these myotubes MyoD levels were reduced (Fig. 1, f–j). Supplying nutrients and serum for 15 h reversed the process. This was illustrated by the cytoplasmic relocalization of MAFbx and by high levels of nuclear MyoD as in control myotubes (Fig. 1, k–o). These observations suggest that MAFbx nuclear translocation is tightly linked to MyoD degradation in muscle cells undergoing atrophy. This hypothesis was strengthened by the fact that ectopic expression of MAFbx-GFP into C2C12 myotubes revealed myonuclear accumulation of the fusion protein and an atrophic phenotype. MyoD staining was lost in these transfected myotubes while C2C12 myotubes transfected with the empty vector were unaffected (Fig. 2). These data show that in muscle cells undergoing atrophy MAFbx is preferentially observed in the nucleus of muscle cells.

### Degradation of MyoD in myotubes undergoing atrophy

MyoD is critical in muscle development and differentiation but MyoD has recently been shown to increase in muscle fibres with maturity in mice and is not due to satellite cell activation [14]. Although MyoD is clearly required for efficient muscle repair [12] the loss of *myoD* function was shown to lead to failure to maintain myonuclear density and increased nuclear domain size in fibres during murine age-related muscle atrophy [14]. We previously showed that the level of MAFbx protein increased in skeletal muscle during aging and/or food deprivation. Immunoprecipitation of the SCF^MAFbx^ complexes from mouse atrophic muscles exhibited ubiquitination activity by using MyoD as substrate [8].

**Figure 3:**
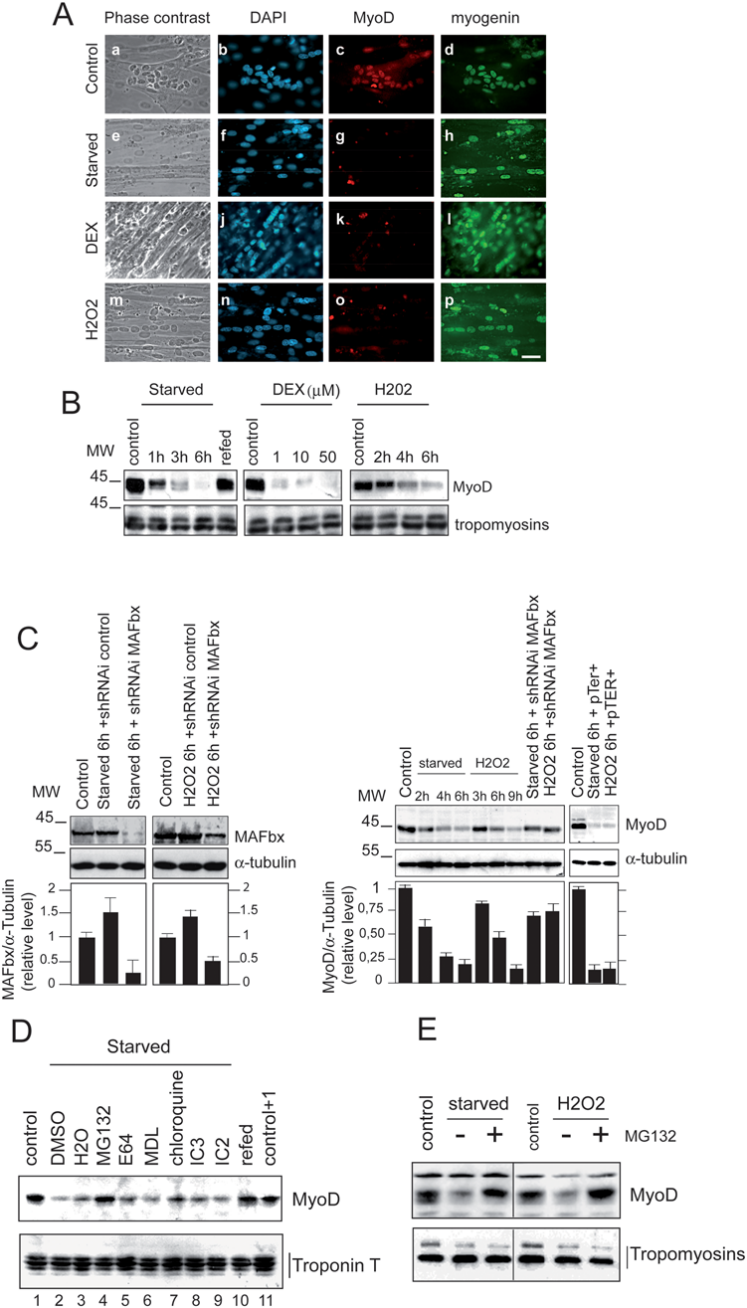
Specific degradation of MyoD during C2C12 myotubes atrophy. A/ Coimmunostaining analysis of MyoD and myogenin in myotubes undergoing atrophy. Myotubes at day 4 of differentiation were treated as described in [7]. Myotubes were fixed and stained with anti-MyoD and anti-myogenin and revealed with goat anti-Rabbit antibody conjugated to Texas Red (c, g, k and l) or with goat anti-mouse antibody conjugated to FITC (d, h, i and p) respectively. DAPI staining shows nuclei (b, f, j and n). Images of a representative field were obtained by indirect immunofluorescence microscopy. (Magnifications are ×400). B/ Proteins were extracted from treated myotubes and subjected to immunoblot analysis with specific anti-MyoD and anti-Tropomyosin antibodies respectively. C/ Depletion of MAFbx by shRNAi prevents MyoD degradation in C2C12 myotubes undergoing atrophy. C2C12 myotubes were transfected by pTER+MAFbx or pTER+control at day 2 of differentiation. Forty-eight hours later myotubes were treated with H_2_0_2_ (225 mM) or were incubated in PBS (starved) and myotubes were harvested 6 h later. Cell lysates were prepared and total proteins were subjected to western blot with anti MyoD, anti MAFbx and anti-α-tubulin antibodies. The graph represents averaged densitometric quantification of the data from three replicate experiments. *P<0.05. D/ Analysis of different cellular proteolysis pathways inhibitors on MyoD protein level during starvation-induced atrophy. C2C12 myotubes at day 4 of differentiation were starved by removal growth medium, amino acids and glucose (starved) for 5 h in the presence of DMS0 (lane 2), H_2_O (lane 3), 30 µM MG132 (lane 4), 30 µM E64 (lane 5), 50 mM MDL (lane 6) 100 µM chloroquine (lanes 7), 10 mM IC3 (lane 8), 25 mM I2C (lane 9) or refed with fresh medium (lane 10). Cell lysates were prepared 5 h later and 50 µg of total proteins were subjected to western blot with anti–MyoD antibodies (Upper panel). Blot was stripped and reprobed with anti-Troponin T antibodies. E/ Myotubes at day 3 of differentiation were treated with H_2_0_2_ (9 h) and/or were starved by removal of growth medium and incubated in PBS (6 h) in the absence (−) of MG132 or in the presence of 30 µM MG132. (+). Fifty micromgramms of total proteins were subjected to western blot with anti–MyoD and anti-Tropomyosin antibodies.

The nuclear localization of MAFbx during muscle atrophy and the loss of MyoD immuno-staining lead us to investigate whether MyoD could be targeted by MAFbx in response to different experimental conditions previously shown to induce atrophy of C2C12 myotubes. Dexamethasone treatment, oxidative stress and starving cultured C2C12 myotubes undergo atrophy by activating MAFbx RNA expression [5], [18]–[20]. Indeed treatment with dexamethasone (DEX), food deprivation (starved) and oxidative stress (H_2_O_2_), all induced a distinct atrophic phenotype characterized by a decrease of∼50–60% in myotubes diameter [7] and an increase of 2–4 fold in MAFbx mRNA and protein levels in response to these experimental conditions. The changes observed in starving myotubes were reversed by resupplying nutrients and serum for 15 hrs (Supplementary data Fig. S2 A to D) [6]. We determined whether in these experimental conditions MAFbx affected MyoD expression. We first examined by immunofluorescence the presence of MyoD in C2C12 myotubes undergoing atrophy. Addition of DEX, starvation and /or oxidative stress, all suppressed MyoD while expression of myogenin was still observed in C2C12 myotubes undergoing atrophy (Fig. 3A). Western blot analysis confirmed the degradation of MyoD in C2C12 myotubes undergoing atrophy. Loss of MyoD by starvation was completely reversed by supplying serum and nutrients . Western blot data also revealed that food deprivation was more efficient in MyoD degradation than the oxidative stress (Fig. 3B). In differentiated myotubes, the cytokine tumor necrosis alpha (TNF-α) plus interferon-gamma (IFN-γ) signalling is required for NF-κB-dependent down regulation of MyoD mRNAs and dysfunction of skeletal myofibrils [21]. To exclude such a mechanism of MyoD regulation, semi-quantitative RT-PCR experiments were undertaken. The results showed that increasing concentration of DEX, starvation and oxidative stress had no significant effect on the expression of MyoD mRNAs (Supplementary data Fig. S2, panel C). Moreover, we used small hairpin RNA interference (shRNAi) in atrophying C2C12 myotubes to assess the role of MAFbx in MyoD suppression. Knockdown of MAFbx in these myotubes prevented by more than 50% the degradation of MyoD while a control shRNAi did not impair its degradation (Fig. 3C). Altogether these results show that activation of MAFbx expression suppresses MyoD in C2C12 myotubes.

### Food deprivation and oxidative stress–induced atrophy increase polyubiquitination by the SCF^MAFbx^ pathway and degradation of MyoD by the proteasome

In order to clear up the proteolytic pathway(s) involved in MyoD degradation during C2C12 myotubes atrophy, we examined the effect of different proteases inhibitors on the amount of MyoD in this process. Addition of cystein protease inhibitor E64, chloroquine, an inhibitor of lysosomal proteolysis, MDL and I2C (inhibitor 2 of calpains) inhibitors of calpains, or IC3, caspases inhibitor III, was ineffective in preventing the degradation of MyoD. On the other hand, treatment with the proteasome inhibitor MG132 conduced to MyoD protein accumulation in myotubes undergoing atrophy (Fig. 3, panels D and E). These data defined the proteasome as the main pathway responsible for MyoD proteolysis during *in cellulo* atrophy.

**Figure 4:**
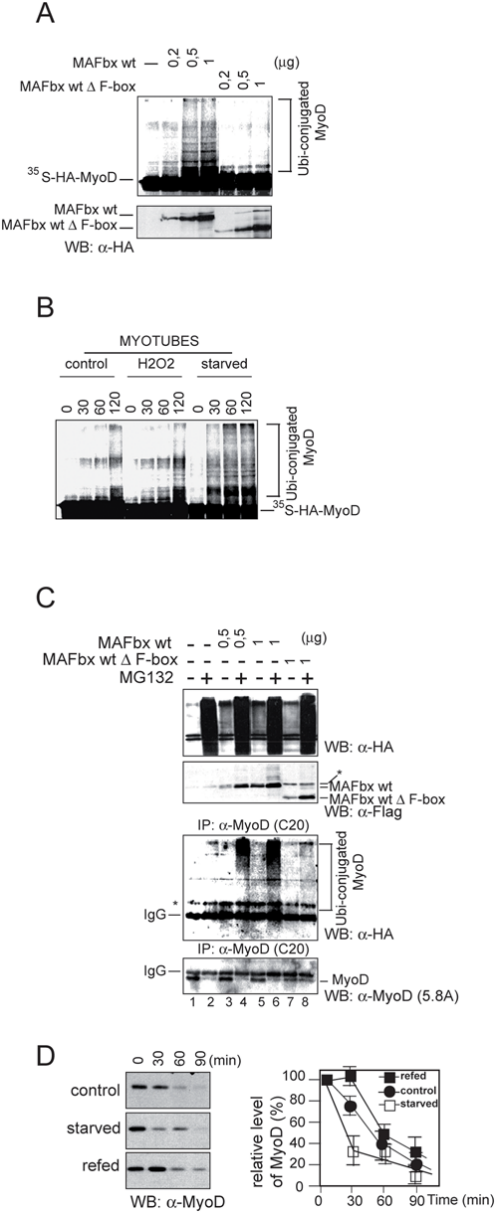
MAFbx promotes polyubiquitination of MyoD *in vitro* and *in cellulo.* A/ MAFbx promotes polyubiquitination of MyoD *in vitro*. ^35^S-labeled MyoD was incubated with E1, ATP, ubiquitin and cdc34 (E2) in the presence of increasing amounts of HA-tagged MAFbx or its F-box deletion mutant produced in rabbit reticulocyte extracts. Reaction mixtures were separated on SDS-PAGE, followed by autoradiography (upper panel) and analyzed by Western blotting with anti-HA antibodies (lower panel). B/ Atrophic myotubes lysates promotes polyubiquitination of MyoD *in vitro*. Myotubes at day 4 of differentiation were treated with H_2_0_2_ (9 h) or were starved for 6 h. Cell lysates were prepared and 30 µg of total proteins were used for *in vitro* ubiquitination assay on equivalent amounts of ^35^S-labeled MyoD. Reaction mixtures were separated on SDS-PAGE and followed by autoradiofluorography. C/ MAFbx increases MyoD ubiquitination *in cellulo*. C2C12 myoblastes were transiently transfected with expression plasmids encoding HA-ubiquitin, Flag-MAFbx wt or the F-box mutant Flag-MAFbx ΔF-box. Transfected cells were treated 3 hours with 30 µM MG132 (+) before harvesting. Cells were lysed in denaturation buffer containing 0.25%SDS. Aliquots (10%) were analyzed by Western blotting with anti-HA and anti-Flag antibodies (upper panels). Cell lysates were then diluted in immunoprecipitation buffer, subjected to immunoprecipitation with a polyclonal anti–MyoD antibody (C-20), and analyzed by Western blotting with anti-Flag antibodies and monoclonal anti-MyoD (5.8A) (lower panels). D/ Increasing instability of MyoD in atrophic C2C12 myotubes. Myotubes at day 4 of differentiation were starved for 6 h. Medium was replaced in refed cultures for 15 h. Myotubes were incubated in methionine-free media for 1 h and then pulsed with 300 µCi/ml of (^35^S)–labeled methionine for 1 h. Following this incubation, cells were washed with media containing an excess of cold methionine (10 mM) and chased for the indicated time. Cell lysates having the same radioactive counts were subjected to immunoprecipitation with the anti-MyoD antibodies (C-20). MyoD was analyzed by SDS-PAGE, followed by autoradiofluorography.

The formation of ubiquitin-protein conjugates involves three components that participate in a cascade of ubiquitin transfer reactions: an ubiquitin-activation enzyme (E1), an ubiquitin conjugating enzyme (E2) and the ubiquitin ligase (E3) that acts at the last step [22]. The association of MAFbx with the essential Skp1, Roc1 and Cul1 proteins, specific components of an E3 ubiquitin-ligase (SCF^MAFbx^) was previously observed in normal mice skeletal muscle and increased amounts were found in skeletal muscle during aging or food deprivation [3], [8]. In *in vitro* ubiquitination assays, SCF^MAFbx^ from recombinant baculoviruses produced in Sf9 cells catalysed the polyubiquitination of MyoD more effectively than the SCF^MAFbx^ from normal skeletal muscle [8]. These data suggest that extend of MyoD ubiquitination is related to MAFbx expression level. To examine this possibility, we first employed a reticulocyte extract system wherein in vitro translated F-box proteins assemble into active SCF [23]. ^35^S–labeled MyoD was incubated with ATP, ubiquitin, ubiquitin activating enzyme (E1) in the presence of increasing amount of MAFbx wt and/or MAFbx-Δ-F-box (produced in reticulocyte extracts) and the ubiquitin conjugating (E2) Cdc34. MyoD was polyubiquitinated in a dose-dependent fashion in the presence of MAFbx wt. In contrast, the F-box deletion mutant MAFbx-Δ-F-box failed to promote polyubiquitination of MyoD (Fig. 4A). Since food deprivation and the oxidative stress lead to rapid myotubes atrophy and large increase in MAFbx expression (Supplementary data , Fig S2), we studied the effects of SCF^MAFbx^ from cellular extracts of atrophic myotubes on MyoD ubiquitination. In *in vitro* ubiquitination assay, the polyubiquitination of MyoD was dramatically increased in atrophic myotubes (Fig. 4B). In addition, in the presence of cellular extracts from starving myotubes, MyoD showed a higher degree of polyubiquitination than with cellular extracts from myotubes treated with H_2_0_2_. This increase in MyoD polyubiquitination probably reflected the fastest degradation of MyoD in starving myotubes (Fig. 3B).

To confirm *in cellulo* the effect of MAFbx overexpression on MyoD polyubiquitination, increasing amounts of expression vectors encoding MAFbx wt or the mutant MAFbx ΔF-box were cotransfected with HA-ubiquitin into C2C12 cells. Cells were collected, lysed in an SDS-containing buffer and MyoD proteins were immunoprecipitated with anti-MyoD antibodies. Immunoprecipitates were then probed with anti-HA to detect ubiquitinated MyoD proteins. In the presence of MAFbx wt, MyoD showed a dose-dependent polyubiquitination (Fig. 4C, lanes 3 and 5), which was increased by MG132 (lanes 4 and 6) while the mutant ΔF-box did not promote ubiquitination of MyoD (lanes 7 and 8). Thus MAFbx induces MyoD polyubiquitination in atrophic myotubes, a function that requires the F-box motif. Finally, as polyubiquitination is now known to be involved in several cellular processes that do not imply proteasomal degradation, including control of transcription factor activity [24], [25], we examined whether MAFbx increased MyoD turnover in atrophic myotubes. To this end, we monitored MyoD protein stability after ^35^S-methionine pulse-labeled of normal and starved myotubes. In untreated myotubes, the half-life of MyoD was 50–55 minutes, a value closed to that found in myoblasts and decreased to 20–30 minutes in starved myotubes (Fig. 4D). A value closed to 30 minutes was also observed for MyoD half-life in H_2_O_2_ treated myotubes (data not shown). These data demonstrate that the rate of MyoD degradation is increased in myotubes undergoing atrophy. Altogether these findings indicate that during *in cellulo* muscle atrophy, MAFbx overexpression increases MyoD polyubiquitination leading to reduction of its half-life.

### Exogenous MyoD delays starvation-induced atrophy in mouse primary myotubes

**Figure 5:**
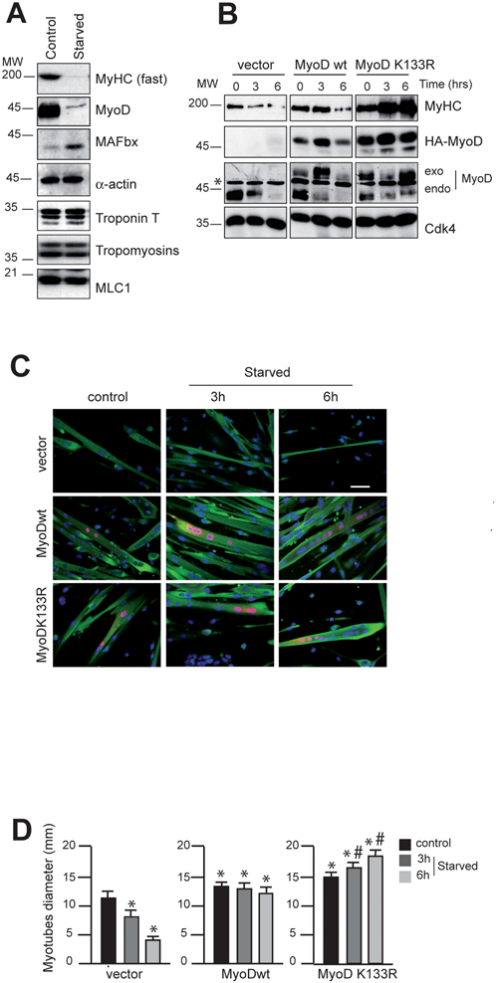
Overexpression of MyoD delays starvation-induced atrophy in primary mouse skeletal muscle myotubes. A/ Primary muscle skeletal myotubes at day 4 of differentiation were starved by removal growth medium, amino acids and glucose and were incubated in PBS for 6 h. Cell lysates were prepared and 50 µg of total proteins were subjected to Western blot analysis to probe for MAFbx, MyoD and myofibrillar proteins. B/ Primary culture of satellite cells were transfected with the expression vector encoding HA-MyoDwt and/or the mutant HA-MyoD K133R as indicated. Twenty-four hours later, cells were homogenized, and at confluence, cells were placed in differentiation medium. Myotubes at day 4 of differentiation were harvested at various times as indicated. Cell lysates were prepared and 50 µg of total proteins were subjected to Western blot with anti–HA antibodies, anti-MyoD antibodies, anti-MyHC antibodies and anti-Cdk4 antibodies as internal control of loading. C/ Myotubes were stained with Hoechst 33258 to visualize the nuclei and with anti-HA antibodies to detect exogenous MyoD expression protein (red) and anti-MyHC antibodies (green) respectively. Images of a representative field were obtained by light microscopy (Scale bar 100 µm). D/ Myotubes diameter after MyoD overexpression in normal and atrophic medium. Experiments were repeated three times with similar results. Data represent the average ±s.e. of at least 150 myotubes. *P<0.05 between control and MyoDwt or MyoD K133R overexpressing cells, ^#^ *P*<0.05 between MyoDwt and MyoD K133R.

In one hand, MyoD has been shown to drive the transcription of Myosin Heavy Chain (MyHC) [26]. On the other hand, MyHC is a preferred target of multiple pro-cachectic factors inducing muscle wasting *in vitro* and *in vivo* [27]. Previous works using the C2C12 culture system demonstrated that TNFα induces an early expression of MAFbx [20] and in combination with IFNγ caused a pronounced reduction of MyHC without affecting the viability the myotubes [21], [28]. This prompted us to test firstly whether MyHC is degraded in starvation-induced mouse primary myotubes atrophy. We observe that in these conditions, MyHC expression is dramatically decreased whereas no appreciable quantitative loss occurred with any of the other core myofibrillar proteins such as Troponin T, Tropomyosins (α and β), α-actin or Myosin Light Chain 1 (MLC1) (Fig. 5A). Secondly we examined whether increasing amounts of MyoD are sufficient to oppose to the loss of MyHC. To test this, we transfected mouse primary myoblasts with expression vectors encoding HA-tagged MyoDwt or MyoDK133R, a mutant insensible to SCF^MAFbx^ polyubiquitination [8]. Twenty-four hours later, cells were homogenized and incubated in differentiation medium for three days. Then, the effects of starving primary cultured myotubes on MyHC protein expression were studied. In this experiment, transfection by the empty vector had no effect on MyHC degradation rate. In contrast, MyoDwt or MyoDK133R overexpression delayed the starvation-induced atrophy and the loss of MyHC (Fig 5B). Interestingly, we also noted that the fibres expressing MyoDK133R displayed a hypertrophic phenotype (Fig. 5C and 8D). Thus, counteracting MyoD degradation protects against *in cellulo* muscle atrophy and MyoDK133R mutant resistant to MAFbx reveals a non-negligible therapeutic interest.

**Figure 6:**
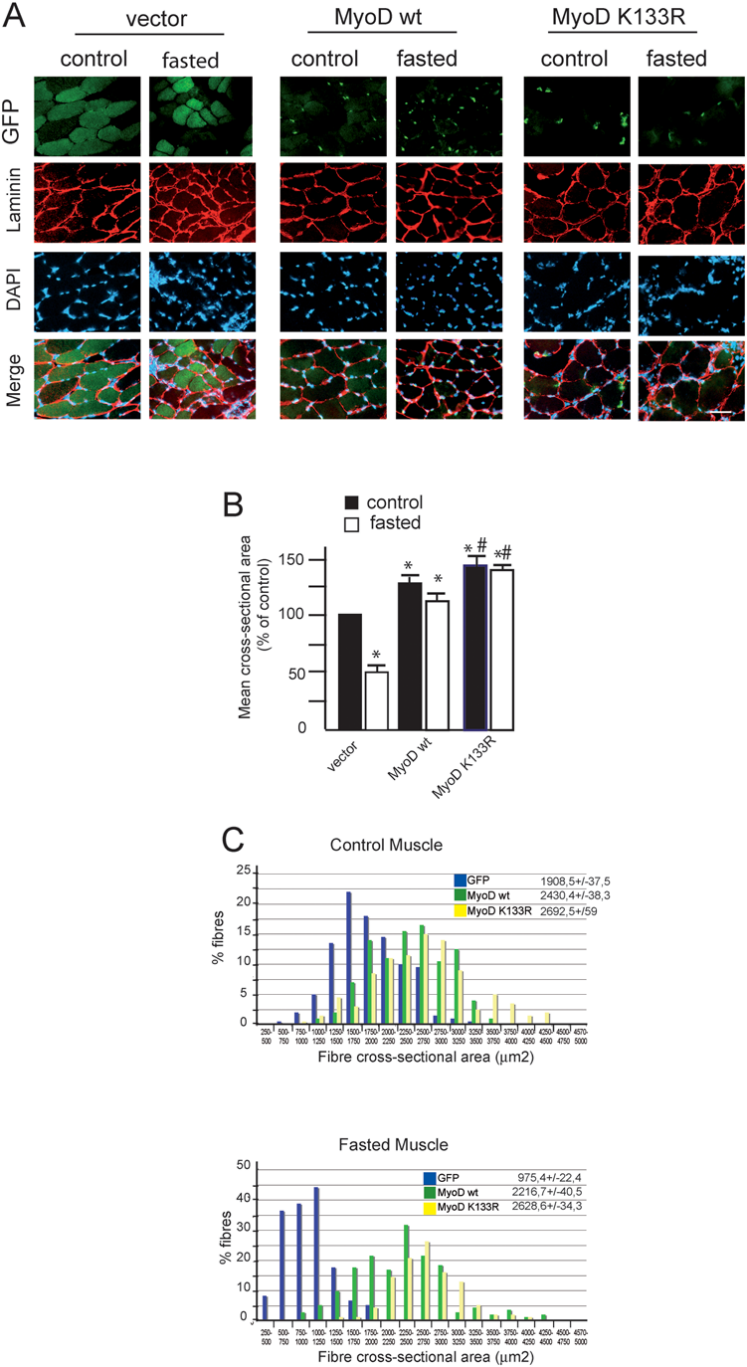
Expression of MyoD K133R in normal and fasted muscle fibres induces hypertrophy. A/ Representative cross-sectional areas of electroporated TA muscles from fed and starved mice. Adult *Tibialis anterior* (TA) muscles were electroporated with either a control (GFP), a MyoDwt-GFP or a MyoD mutant K133R-GFP plasmid. Mice (control or fasted by 2 days) were sacrificed 14 days later. Transfected fibres were identified on the basis of their GFP expression. The distribution of cross-sectional areas of EGFP-expressing fibres taken from four muscles under each conditions Fibres membranes were stained with polyclonal anti-laminin antibody. Scale bar, 20 µm. B/. Mean cross-sectional area of TA fibres in control (fed) and fasted mice (atrophy)±s.e.m. *P<0.05 between control and MyoDwt or MyoD K133R electrotransferred fibres. # P<0,05 between MyoDwt and MyoD K133R. n>200 fibres. C/. Histogram shows the fibre size distribution of TA from four mice per expression vector (in control: fed and atrophy: fasted mice). Blue bars, empty vector, green bars MyoDwt and yellow bars mutant MyoD K133R.

To extend our findings *in vivo*, we electroporated expression vectors encoding MyoDwt or MyoDK133R bound to Enhanced Green Fluorescent Protein (EGFP) into the *Tibialis anterior* (TA) muscle of the right hind leg and the EGFP vector into the left hind leg as an internal control. With this electroporation technique only muscle fibres express EGFP, satellite cells are not electroporated (Fig. 6A and L. Schaeffer, personal communication). Fourteen days after electroporation, mice were either starved for 48 h (starved) or allowed free access to food (control). In four EGFP, four MyoDwt-EGFP and four MyoDK133R-EGFP treated normal TA muscles, cross sectional area was determined in fibres expressing EGFP (about 200 fibres per muscle). Mean fibre size was significantly larger in fibres overexpressing MyoDwt (2430.4+/−38.3 µm^2^) and MyoDK133R (2692.5+/−59 µm^2^) than in fibres overexpressing the control EGFP alone (1908.5+/−37.5 µm^2^). The distribution of fibre sizes shifted to the right, with the range in fibre sizes increasing from 711 to 3530 µm^2^ in control EGFP muscles to 1184 to 3621 µm^2^ in muscles overexpressing MyoDwt and to 867 to 5334 µm^2^ with MyoDK133R. Then, the MyoD electroporated fibres in control mice showed a hypertrophic phenotype notably characterized by a 47% increase in mean cross-section area (CSA) for MyoDK133R (Fig. 6B). Forty-eight hours starvation reduced CSA by 48.9% in fibres expressing EGFP alone (975.4+/−22.4 µm^2^) whereas CSA from MyoDwt-electroporated fibres showed only an 11.2% decrease (2216.7+/−40.5 µm^2^). Of note, hypertrophic fibres expressing MyoDK133R were completely resistant to food deprivation (2628.6+/−34.3 µm^2^) (Fig. 6, B and C). These data demonstrate for the first time that maintaining MyoD expression in fibre myonuclei plays a key and unsuspected role in preventing starvation-induced skeletal muscle atrophy *in vivo*.

## Discussion

We have identified MyoD as the second MAFbx target in skeletal muscle atrophy. Depending on the atrophic signaling pathway, MyoD expression seems to be inhibited both at the RNA and protein levels suggesting that *in vivo*, MyoD down-regulation is crucial for muscle loss [8], [21], [29]. On one hand, whether MAFbx could participate in the muscle wasting process *in vivo* by impairing satellite cells proliferation and differentiation through MyoD degradation remains to be determined. On the other hand, in fibre myonuclei, MyoD function is not elucidated. MyoD expression seems necessary to maintain or to drive muscle-specific gene transcription like *MyHC* in response to hypertrophy, denervation or exercise [15], [16]. It has also been reported that when skeletal muscles undergo atrophy, a subset of myonuclei is lost by apoptosis even though the rest of the fibre is not affected by this cellular death. It has been proposed that this process helps muscle fibres to carefully maintain the cytoplasmic domain controlled by a single myonucleus [30], [31]. Of note, loss of *myoD* function leads to failure to maintain this myonuclear density, a mechanism potentially linked to satellite cells differentiation defects [14]. Moreover MyoD directly transactivates the cyclin-dependent kinase inhibitor p21 (p21) and the retinoblastoma protein (Rb), a downstream target of p21 *in vitro*. These factors act to regulate cell cycle withdrawal and antiapoptotic cell death [32]. Hence, MyoD could also control antiapoptotic pathways inside the fibre to maintain muscle homeostasis.

In addition, skeletal muscle atrophy is characterized by enhanced myofibrillar destabilization and proteolysis [33]. However, to date, MAFbx does not seem to target core skeletal myofibrillar proteins. While in the cardiomyocytes, MAFbx has been shown to interact with calcineurin A and α-actinin 2 at the Z-disc [17], in skeletal muscle cells, MAFbx overexpression did not modify calcineurin A protein levels nor interact with α-actinin 2 (data not shown). We propose that MAFbx most direct function in the atrophic process is to act in parallel to myofibrillar proteolysis. Our results strongly suggest that MAFbx overexpression in atrophic muscle fibres not only inhibits skeletal muscle protein translation via eIF3-f proteolysis [7] but also acts through suppression of MyoD specific transcriptional activity among the fibre. Impairment of these two pathways is likely to conduce to muscle fibre shift from anabolism to catabolism. However, if MAFbx-dependent activation of Foxo transcription factors discovered in cardiomyocytes [24] is conserved in skeletal muscle fibres, MAFbx would also play an indirect role on skeletal myofibrillar proteolysis stimulation.

The eventual outcome of skeletal muscle atrophy studies is to develop efficient muscle-specific strategies to prevent and/or slow down skeletal muscle loss. The new data presented here suggest that designing MAFbx specific inhibitors that disrupt its interaction with MyoD or overexpressing MyoDK133R in muscle fibres would help to achieve this goal.

## Materials and Methods

### Plasmids constructions

Expression vectors encoding HA and/or Flag tagged MAFbx wt and MAFbx Δ-F-box, HA-tagged MRFs and HA-ubiquitin were previously described [8], [34]. ShRNAi sequence (shRNAi mouse MAFbx, 5′-GATCCCCCAGAAGATTCAACTACGTTTCAAGAGAACGTAGTTGATCTTCTGGTTTTTGGAAA-3′) was incorporated into 64 bp self-annealing oligonucleotides and cloned into pTER+ expression vector (pTER+-MAFbx), (a gift of Van de Wetering, Hubrecht Laboratory Center for Biomedical Genetics, Utrecht, The Netherlands).

### Cell culture and Transfections

The mouse skeletal muscle cell line C2C12 and the mouse fibroblastic 10T1/2 cells ere grown in Dulbecco's modified Eagle's medium (DMEM) supplemented with 20% of fetal calf serum (FCS) and antibiotics. Myoblast fusion and differentiation was induced in subconfluent cells by replacing the medium with DMEM 2% FCS. Primary cultures were prepared from male mice from our own breeding stocks. All animals were treated in accordance with institutional and national guidelines. Mice satellite cells were isolated from the whole muscles of the paw. Cells were plated at a density of 2×10^4^ cell/cm^2^ on Matrigel-coated Petri dishes (BD Biosciences), in 80% Ham's-F10 medium containing glutamine, penicillin and amphotericin B (Invitrogen), supplemented with 20% horse serum. After two days, cells were washed with Ham's-F10 and placed in complete medium supplemented with 5 ng/mL basic fibroblast growth factor. Atrophy was induced in cultured myotubes by switching the medium to PBS (100 mM NaCl, 5 mM KCl, 1.5 mM MgSO_4_, 50 mM NaHCO_3_, 1 mM NaH_2_PO_4_, 2 mM CaCl_2_) for 6 hours. Primary cultures of satellite cells were transfected with 2 µg of total plasmid using Dreamfect (OZBiosciences). C2C12 myoblasts were cultured in 36-mm dishes and transfected with 3 µg of total plasmid by using Lipofectamin 2000 (Invitrogen). High-level transfection efficiency for C2C12 myoblasts was achieved by using a modified protocol for Lipofectamin 2000. Freshly trypsinized myoblasts were transfected 30 min after plating (300×10^3^ cells/36-mm dish) with a 2∶1 ratio (µl /µg) of Lipofectamin 2000 (10 µl/36-mm dish) to plasmid DNA (5 µg/36-mm dish). An expression vector coding for the GFP protein was transfected as control of transfection efficacy.

### Immunoprecipitation and immunoblotting

An anti-MAFbx antibody was generated by injecting rabbits with a GST-MAFbx fusion protein corresponding to aa 1–102 of the human MAFbx protein. Antibodies were affinity purified against an MBP-MAFbx fusion protein. Myotubes were rinsed in cold PBS and lysed in IP buffer (50 mM Tris pH 7.4, 150 mM NaCl, 10% glycerol, 0.5% NP40, 0.5 mM Na-orthovanadate, 50 mM NaF, 80 µM β-glycerophosphate, 10 mM Na-pyrophosphate, 1 mM DTT, 1 mM EGTA and 10 µg/ml leupeptin, 10 µg/ml pepstatin and 10 µg/ml aprotinin). Lysates were precleaned for 30 min with protein-G beads and immunoprecipitated by using standard procedures. Immunoprecipitated proteins were loaded onto 10% SDS/PAGE gels before electrophoretic transfer onto nitrocellulose membrane. For detection of MyoD-ubiquitin conjugates, cells were rinsed in phosphate buffer saline and scrapped in RIPA buffer (20 mM Tris, pH 7.5, 5 mM EDTA, 150 mM NaCl, 1% NP40, 0.5% Na-deoxycholate, 0.025% SDS, 1 mMNa-orthovanadate, 10 mM NaF, 25 µM β-glycerophosphate and 1 µM leupeptin, 1 µM pepstatin and 10 µM aprotinin). Cells were then spun at 15000×g for 15 min and the supernatant was precipitated with anti-MyoD antibody. Western blotting was performed by using an ECL kit (Amersham) according to the manufacturer's instructions. Anti MyoD polyclonal (C20) was purchased from Santa Cruz Biotechnology, Anti-Troponin T monoclonal (JLT-12) and anti-tropomyosin (TM 311) were from Sigma, anti-HA epitope (12CA5) was purchased from Roche, anti-MyoD monoclonal (5.8A) and anti-myogenin (F5D) were from Pharmingen and anti-Flag (M2) was from Sigma. To inhibit proteasome activity, cells were treated with 30 µM of MG132 (Sigma) for 3 h.

### Protein extraction for *in vitro* ubiquitination

C2C12 myotubes were suspended in 1,5 volume of ice-cold buffer consisting of 25 mM Tris-HCl (pH 7.4), 2 mMDTT, 0,25 mM EDTA, 10 µg/ml leupeptin, 10 µg/ml pepstatin and incubated in ice for 15 min. After addition of 0,2% Triton ×100 for 10 min the lysate was transferred to an Eppendorf tube and centrifuged in a microcentrifuge at 10,000 g for 10 min. The supernatant was divided into smaller samples and frozen at −80°C.

### *In vitro* and *in vivo* ubiquitination assay

Ubiquitination assays were determined by using (^35^S) methionine–labelled *in vitro* translated MyoD [8], [35]

### RNA extraction and RT-PCR analyses

Total RNA was isolated from C2C12 cells using TRI REAGENT (Sigma). For semi quantitative RT-PCR analysis, 1 µg of RNA was used to perform reverse transcription with Superscript II RNaseH Reverse Transcriptase and oligodT (Invitrogen). Two microliters of each reaction served as a template for PCR analysis. Primer pairs for the amplification of the gene products were the following: *MAFbx* 5′-GGGGGAAGCTTTCAACAG-3′ forward, 5′TGAGGCCTTTGAAGGCAG-3′ reverse, MyoD 5′-CCCGGCGGCAGAATGGCTACG-3′forward, 5′-GGTCTGGGTTCCCTGTTCTGTG-3′ reverse and *GAPDH* 5′-GAGCTGAACGGGAAGCTCACT-3′ forward, 5′-TTGTCATACCAGGAAATGAGC-3′ reverse.

### Immunofluorescence staining and Microscopy

Cells were cultured on coverslips and fixed in 3% Para formaldehyde for 30 min at 4°C, and permeabilized with 0.5% Triton X-100 for 30 min at room temperature. The cells were treated with 5% normal goat serum and immunostained with the mAb anti-MyoD (5.A8) and/or the polyclonal anti-MyoD (C-20), anti-myogenin (F5D) and/or the purified anti-MAFbx. The Texas red-conjugated F (ab')_2_ fragments of donkey anti-mouse IgG were used to visualize the mouse monoclonal antibodies and the FITC-conjugated F(ab')_2_. Fragments of Donkey anti-rabbit IgG to visualize the rabbit polyclonal antibodies.

Cells were rinsed in PBS containing DAPI (Sigma), mounted in citifluor and then were examined on a phase-contrast microscope Axioplan 135 M; Zeiss with a 40× oil immersion objective at room temperature. Images were captured with a camera model (Axiocam HRc, Zeiss) using Axiovision software Rel.4, Zeiss) and Photoshop 8.0 (Adobe).

Confocal microscopy was performed with primary mouse skeletal muscle myoblasts. Cells grown on polylysine-coated cover lips were fixed in 3% paraformaldehyde/2% sucrose in PBS at pH 7.4 for 15–20 minutes at 37°C. After three washes in PBS, cells were permeabilized using 0.2% Triton X-100 in PBS for 5 minutes at room temperature. Covers lips were rinsed three times with PBS and incubated for 1 h at room temperature with the indicated primary antibody diluted in PBS containing 1% BSA and 1% goat serum. After three washes in PBS, the appropriate secondary antibody was used. Myosin heavy chain (MHC) was detected using a mouse monoclonal anti-MHC (My32)from Sigma) and stained with anti mouse Texas-Red (Jackson Immunoresearch Inc. (West Grove, PA). DNA was counterstained with 1 µg ml^−1^ TOPRO-3 (Molecular Probes). Covers lips were then inverted into Vectashield medium (Vector Lab. Burlingare, CA). Images were acquired on a laser confocal microscope Zeiss LSM 510 Meta system with a 40× objective and processed with Image VisArt and Photoshop 8 (Adobe).

### Muscle electro transfer

Ethical approval was obtained for the use of animals in our study (Ministère de l'Enseignement supérieur et de la Recherche , N° 4962, 12/03/2008) .All animals experiments were performed according to European directives (86/609/CEE). In vivo transfection experiments were carried out on four 8-week-old C57BL/6 females. Mice were first anesthetized with isoflurane (0,75% to 1% in oxygen) and received a single injection of 0,4 U of bovine hyaluronidase (SIGMA)/ml in 25 ml 0,9% NaCl into the Tibialis anterior (TA) muscle. 2 hours after, a total of 15 mg of plasmid DNA in 0,9% NaCl was injected into the TA under conditions of ketamine (100 µg/g of body weight) and xylazine (10 µg/g) anaesthesia. pCMV-EGFP was used as a control and injected in the left leg while pCMV-EGFP-MyoDwt or pCMV-EGFP-MyoD K133R was injected in the contra lateral muscle. An electrical field was then applied to muscle with calliper rule electrodes coated with ultrasound transmission gel (Aquasonic 100, Parker) and placed on each side of the leg. Six square-wave 130 V/cm pulses, lasting 60 ms each with a 100 ms interval, were then applied with a BTX electrocell manipulator. Mice were killed by cervical dislocation, and muscles were collected 14 days after electro transfer.

### Histological analysis and fibre measurement

Fasted (48 h) and control (fed) C57BL/6 mice were killed by cervical dislocation. *Tibialis Anterior* (TA) muscles were removed, embedded in cryomatrix and quickly frozen in isopentane cooled with liquid nitrogen. Muscles were then sectioned in a microtome cryostat (Leica). Transversal sections (12 µm) were fixed with methanol for 5 min at −20°C, rinsed three times with PBS, permeabilized with 0.1% Triton X-100 and blocking 1 h (3% BSA, 20% NGS in PBS). Rabbit polyclonal anti-Laminin antibody (Sigma) was applied overnight to the treated sections. Bound primary antibodies were detected with cyanine 3-conjugated goat anti-rabbit Texas Red IgG (Jackson Immunoresearch). Nuclei were stained with Hoechst. Fibre cross-sectional areas were measured using Perfect Image software and determined for 4 sections from our animals in each group.

### Statistics

All data are expressed as the mean±SEM. Data were evaluated by one way analysis of variance (ANOVA) followed by Tukey's Honestly Significant Differences test (SigmaSTAT Software). A *P*-value of <0.05 was considered statistically significant.

## Supporting Information

Figure S1 Specific interaction of MyoD with MAFbx. 10T1/2 fibroblastic cells were cotransfected with expression vectors encoding HA-tagged MyoD, HA-tagged myogenin, HA-tagged MRF4 or empty vector together with Flag-tagged MAFbx. Cells were harvested 24 hr after transfection. Aliquots of total cell extracts were directly probed with anti-HA and/or anti-Flag antibodies (Inputs) and 400 µg of total proteins were immunoprecipitated with anti-Flag antibodies prior to Western blotting with anti-HA and anti-Flag antibodies respectively (IP).* non specific band.
(1.88 MB EPS)

Figure S2 Increase expression of MAFbx in myotubes undergoing atrophy. A/ Morphology of normal and atrophic C2C12 myotubes. Myotubes at day 4 of differentiation were treated with 225 µM H202 (9 h), 50 µM dexamethasone (24 h) and/or were starved by removal of growth medium, amino acids and glucose and incubated in PBS for 6 h (staved). Medium was replaced in refed cultures for 15 h (Refed). (Magnifications are ×400). B/ Measurement of average myotubes diameter after treatments as described in (A). Results are presented as the mean(n>100 myotubes per conditions) +/−SEM. C/Analysis of MAFbx and MyoD mRNA expression by semi-quantitative RT-PCR. Myotubes were treated with increasing concentrations of dexamethasone for 24 h (DEX, 1, 10 and 50 µM), or incubated either in PBS for 6 h or in H2O2 (225 µM) for 4 h and 9 h respectively. GAPDH expression was used as an internal control.
(0.72 MB TIF)

## References

[1] Jagoe RT, Goldberg AL (2001). What do we really know about the ubiquitin-proteasome pathway in muscle atrophy?. Curr Opin Clin Nutr Metab Care.

[2] Bodine SC, Latres E, Baumhueter S, Lai VK, Nunez L (2001). Identification of ubiquitin ligases required for skeletal muscle atrophy.. Science.

[3] Gomes MD, Lecker SH, Jagoe RT, Navon A, Goldberg AL (2001). Atrogin-1, a muscle-specific F-box protein highly expressed during muscle atrophy.. Proc Natl Acad Sci U S A.

[4] Cenciarelli C, Chiaur DS, Guardavaccaro D, Parks W, Vidal M (1999). Identification of a family of human F-box proteins.. Curr Biol.

[5] Winston J, Koepp DM, Zhu C, Elledge SJ, Harper JW (1999). A family of F-box proteins.. Curr Biol.

[6] Sandri M, Sandri C, Gilbert A, Skurk C, Calabria E (2004). Foxo transcription factors induce the atrophy-related ubiquitin ligase atrogin-1 and cause skeletal muscle atrophy.. Cell.

[7] Lagirand-Cantaloube J, Offner N, Csibi A, Leibovitch M-P, Batonnet-Pichon S (2008). The initiation factor eIF3-f is a major target for atrogin1/MAFbx function in skeletal muscle atrophy.. EMBO J.

[8] Tintignac LA, Lagirand J, Batonnet S, Sirri V, Leibovitch M-P (2005). Degradation of MyoD mediated by the SCF (MAFbx) ubiquitin ligase.. J Biol Chem.

[9] Edmondson DG, Olson EN (1993). Helix-loop-helix proteins as regulators of muscle-specific transcription.. J Biol Chem.

[10] Kitzmann M, Carnac G, Vandromme M, Primig M, Lamb N (1998). The Muscle Regulatory Factors MyoD and Myf-5 Undergo Distinct Cell Cycle-specific Expression in Muscle Cells.. J Cell Biol.

[11] Tintignac LA, Sirri V, Leibovitch M-P, Lecluse Y, Castedo M, Metivier D (2004). Mutant MyoD lacking Cdc2 phosphorylation sites delays M-phase entry.. Mol Cell Biol.

[12] Megeney LA, Kablar B, Garrett K, Anderson JE, Rudnicki MA (1996). MyoD is required for myogenic stem cell function in adult skeletal muscle.. Genes Dev.

[13] Hughes SM, Koishi K, Rudnicki MA, Maggs AM (1997). MyoD protein is differentially accumulated in fast and slow skeletal muscle fibres and required for normal fibre type balance in rodents.. Mech Dev.

[14] Brack AS, Bildsoe H, Hughes SM (2005). Evidence that satellite cell decrement contributes to preferential decline in nuclear number from large fibres during murine age-related muscle atrophy.. J Cell Sci.

[15] Ishido M, Kami K, Masuhara M (2004a). Localization of MyoD, myogenin and cell cycle regulatory factors in hypertrophying rat skeletal muscles.. Acta Physiol Scand.

[16] Ishido M, Kami K, Masuhara M (2004b). In vivo expression patterns of MyoD, p21, and Rb proteins in myonuclei and satellite cells of denervated rat skeletal muscle.. Am J Physiol Cell Physiol.

[17] Li HH, Kedar V, Zhang C, McDonough H, Arya R (2004). Atrogin-1/muscle atrophy F-box inhibits calcineurin-dependent cardiac hypertrophy by participating in an SCF ubiquitin ligase complex.. J Clin Invest.

[18] Hasselgren PO (1999). Glucocorticoids and muscle catabolism.. Curr Opin Clin Nutr Care.

[19] Stitt TN, Drujan D, Clarke BA, Panaro F, Timofeyva Y (2004). The IGF-1/PI3K/Akt pathway prevents expression of muscle atrophy-induced ubiquitin ligases by inhibiting FOXO transcription factors.. Mol Cell.

[20] Li YP, Chen Y, John J, Moylan J, Jin B (2005). TNF-alpha acts via p38 MAPK to stimulate expression of the ubiquitin ligase atrogin1/MAFbx in skeletal muscle.. FASEB J.

[21] Guttridge DC, Mayo MW, Madrid LV, Wang CY, Baldwin AS (2000). NF-kappaB-induced loss of MyoD messenger RNA: possible role in muscle decay and cachexia.. Science.

[22] Glickman MH, Ciechanover A (2002). The ubiquitin-proteasome proteolytic pathway: destruction for the sake of construction.. Physiol Rev.

[23] Koepp DM, Schaefer LK, Ye X, Keyomarsi K, Chu C (2001). Phosphorylation-dependent ubiquitination of cyclin E by the SCFFbw7 ubiquitin ligase.. Science.

[24] Li HH, Willis MS, Lockyer P, Miller N, McDonough H (2007). Atrogin-1 inhibits Akt-dependent cardiac hypertrophy in mice via ubiquitin-dependent coactivation of Forkhead proteins.. J Clin Invest.

[25] Sun L, Chen ZJ (2004). The novel functions of ubiquitination in signaling.. Curr Opin Cell Biol.

[26] Allen DL, Sartorius CA, Sycuro LK, Leinwand LA (2001). Different pathways regulate expression of the skeletal myosin heavy chain genes.. J Biol Chem.

[27] Acharyya S, Ladner KJ, Nelsen LL, Damrauer J, Reiser PJ (2004). Cancer cachexia is regulated by selective targeting of skeletal muscle gene products.. J Clin Invest.

[28] Ladner KJ, Caligiuri MA, Guttridge DC (2003). Tumor necrosis factor-regulated biphasic activation of NF-kappa B is required for cytokine-induced loss of skeletal muscle gene products.. J Biol Chem.

[29] Langen RC, Van Der Velden JL, Schols AM, Kelders MC (2004). Tumor necrosis factor-alpha inhibits myogenic differentiation through MyoD protein destabilization.. FASEB J.

[30] Liu CC, Ahearn JM (2001). Apoptosis of skeletal muscle cells and the pathogenesis of myositis: a perspective.. Curr Rheumatol Rep.

[31] Tews DS (2005). Muscle-fiber apoptosis in neuromuscular diseases.. Muscle Nerve.

[32] Kitzmann M, Fernandez A (2001). Crosstalk between cell cycle regulators and the myogenic factor MyoD in skeletal myoblasts.. Cell Mol Life Sci.

[33] Ventadour S, Attaix D (2006). Mechanisms of skeletal muscle atrophy.. Curr Opin Rheumatol.

[34] Sirri V, Leibovitch M-P, Leibovitch SA (2003). The muscle regulatory factor MRF4 activates differentiation in rhabdomyosarcoma RD cells through a positive-acting C-terminal protein domain.. Oncogene.

[35] Batonnet S, Leibovitch M-P, Tintignac LA, Leibovitch SA (2004). Critical role for lysine 133 in the nuclear ubiquitin-mediated degradation of MyoD.. J Biol Chem.

