# Supplementary figures and images for: Inhibition of Atrogin-1/MAFbx Mediated MyoD Proteolysis Prevents Skeletal Muscle Atrophy In Vivo
Source: PLoS One. 2009 Mar 25;4(3):e4973. doi: 10.1371/journal.pone.0004973 (PMC2656614; doi:10.1371/journal.pone.0004973)

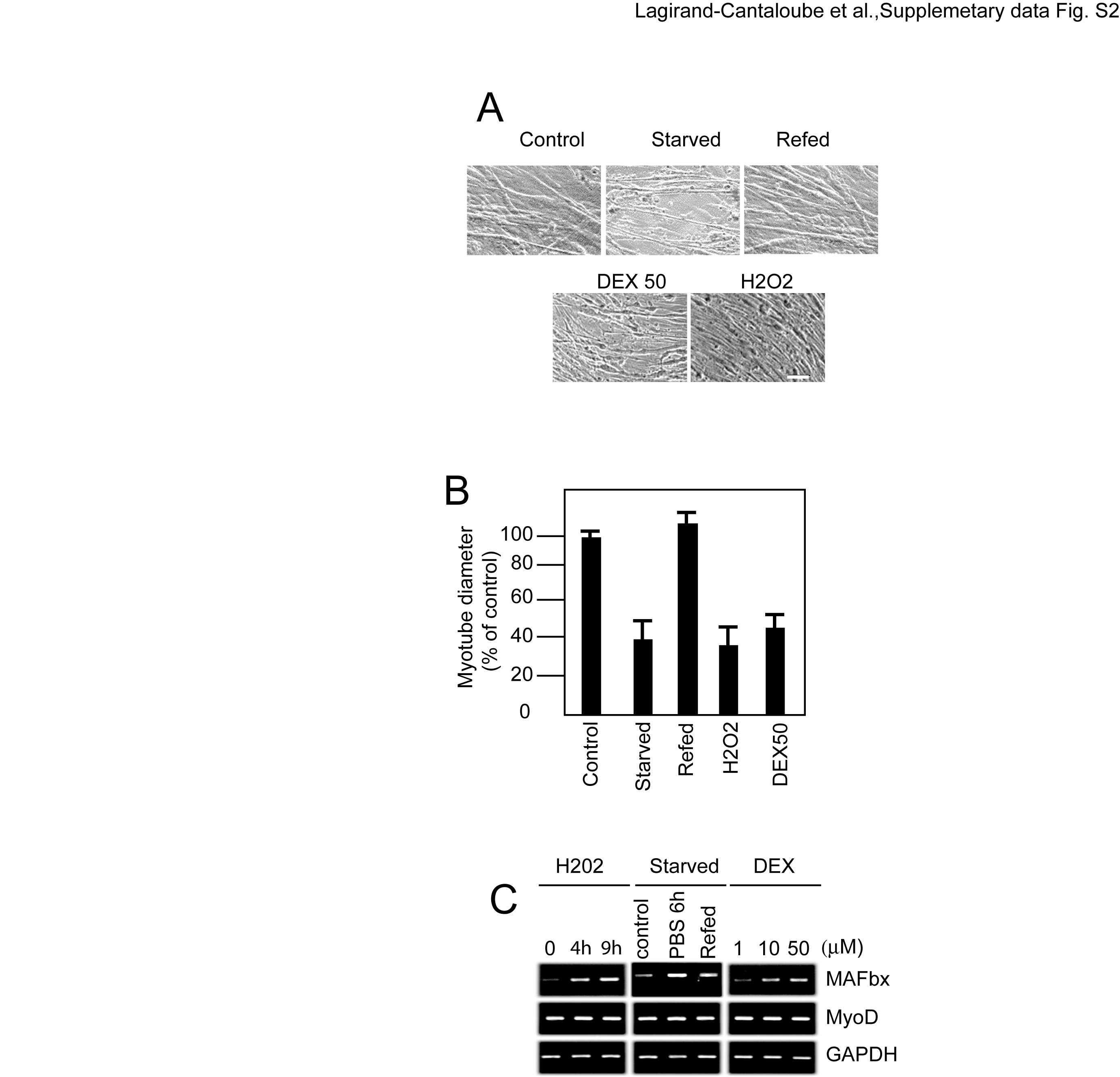

Supplement: Figure S2 — Increase expression of MAFbx in myotubes undergoing atrophy. A/ Morphology of normal and atrophic C2C12 myotubes. Myotubes at day 4 of differentiation were treated with 225 µM H202 (9 h), 50 µM dexamethasone (24 h) and/or were starved by removal of growth medium, amino acids and glucose and incubated in PBS for 6 h (staved). Medium was replaced in refed cultures for 15 h (Refed). (Magnifications are ×400). B/ Measurement of average myotubes diameter after treatments as described in (A). Results are presented as the mean(n>100 myotubes per conditions) +/−SEM. C/Analysis of MAFbx and MyoD mRNA expression by semi-quantitative RT-PCR. Myotubes were treated with increasing concentrations of dexamethasone for 24 h (DEX, 1, 10 and 50 µM), or incubated either in PBS for 6 h or in H2O2 (225 µM) for 4 h and 9 h respectively. GAPDH expression was used as an internal control. (0.72 MB TIF) [file pone.0004973.s002.tif]
